# Quantitative predictions of diverse wrinkling patterns in film/substrate systems

**DOI:** 10.1038/s41598-017-18267-0

**Published:** 2017-12-22

**Authors:** Fan Xu, Michel Potier-Ferry

**Affiliations:** 1 Institute of Mechanics and Computational Engineering, Department of Aeronautics and Astronautics, Fudan University, 220 Handan Road, Shanghai, 200433 P. R. China; 2 Laboratoire d’Etude des Microstructures et de Mécanique des Matériaux, LEM3, UMR CNRS 7239, Université de Lorraine, 7 Rue Félix Savart, 57073 Metz Cedex 03, France

## Abstract

A basic characteristic of stiff film/soft substrate systems is their ability to experience large deformation under compressive stresses, which inevitably leads to formation of patterns on the surface. Such pattern formation is the result of loss of stability and symmetry breaking. Knowledge on how such instabilities arise and evolve is essential to describe, understand, predict, and ultimately to design complex functional materials and structures, for example the fabrication of stretchable electronic devices and micro/nano-scale surface patterning control. In this paper, quantitative predictions of various instability pattern formations and evolutions, which involve highly nonlinear deformation and multiple bifurcations, will be presented based on advanced mechanical models and methods, from planar to curved geometry. The results can provide further insight into fundamental understanding in a whole view of a variety of surface patterning morphology and imply a potential way to facilitate the design of functional materials and structures by quantitatively harnessing surface instabilities.

## Introduction

Surface morphological instabilities of a soft material with a stiff thin surface layer have raised considerable research interests during past few years. Abundant examples can be found in various types of living creatures across length scales such as blooming process of hornbeam leaves^1^, hierarchical wrinkling of skins^2^ and fingers^3^, folding of growing tubular organs^4^ and human brain development^5^, morphological buckling of fruits and vegetables^6–8^, and differential growth of bacterial biofilms^9^. Besides, in modern industry, surface wrinkling can be widely applied in large area ranging from micro/nano morphological patterning control^10–12^, fabrication of flexible electronic devices^13,14^, mechanical self-assembly of islands on nano-particles^15^, defect localization in elastic surface crystals^16^, wet surface chemical patterning of micro-spheres^17^, multi-periodic surface topography of coated materials^18^, adaptive aerodynamic drag control^19,20^, mechanical property measurement of material characteristics^21^, to the design of moisture-responsive wrinkling devices with tunable dynamics^22^ and reversible optical writing/erasure functional surface^23^. These phenomena or functions with patterning morphology involve surface instability and symmetry breaking which are usually induced by large deformation of film/substrate systems under compressive stresses. Knowledge on how such instabilities arise and evolve is essential to describe, understand, predict, and ultimately to design complex functional materials and structures as listed above.

Although linear perturbation analyses can predict the wrinkling wavelength at the initial stage of instability threshold, determining the post-bifurcation response and surface mode transition requires nonlinear buckling analyses. During post-buckling, the wavelength, amplitude and instability mode may vary with respect to external load. Due to its well-known complexity, most recent post-buckling analyses have recourse to computational approaches, especially through finite element method^24–32^, since a limited number of exact analytical solutions can be obtained only in very simple or simplified cases^33^. Nevertheless, surface instability of stiff layers attached on soft materials usually involves strong geometrical nonlinearities, large rotations, large displacements, loading path dependence, multiple symmetry-breakings, nonlinear constitutive relations, localizations and other complexities, which makes the numerical resolution quite difficult^33^. The morphological post-buckling evolution and mode transition beyond the critical load are incredibly complicated, especially in 3D cases, and conventional numerical methods are limited in studying the post-bifurcation response on their complex evolution paths. Besides, several early works in the literature apply Fourier-based methods^34–36^, which prescribe periodic boundary conditions and cannot account for boundary effects on pattern evolution. A general model incorporating reliable and robust resolution methods is in strong demand for post-buckling analyses of film/substrate systems, especially for predicting and tracing the surface mode transition. Inspired by this, we propose a unified 3D model, associated with geometrically nonlinear shell formulations for the surface layer and linear elastic solids for the substrate, to quantitatively investigate the occurrence and post-buckling evolution of a variety of wrinkling patterns in film/substrate systems under various loadings. The model incorporates a robust path-following technique to predict a sequence of secondary bifurcations on their nonlinear equilibrium path as the load increases and provides an overall view of pattern evolution. Such finite element model allows considering all the data of a boundary value problem (geometry, boundary conditions, loading and material properties), and describing their influences on pattern formation and pattern evolution.

Besides, many natural or artificial film/substrate systems have curved geometry. Recent experimental investigations reveal that wrinkling patterns may vary with the substrate curvature^17,19^ and wrinkling processes under curvature constraints become promising techniques for micro/nano-scale surface patterning fabrication^15,37^ and control^20^. Curvature-induced wrinkling pattern formation and selection are only lately being pursued theoretically and numerically^12,24,28,30,38^, which demonstrates the important role of topological curvature constraints on pattern selection in non-planar geometries. Nevertheless, quantitative prediction and tracing of the whole pattern evolution on curved surfaces are still challenging due to strong nonlinearities and geometric complexities, which merit much further investigations. Based on our proposed model, we trace the occurrence and post-buckling evolution of diverse instability modes on non-planar geometry, including churro-like and buckyball-like patterns, and advance the fundamental understanding of the whole view of pattern evolution. The results imply a potential way to facilitate the design of functional materials and structures by harnessing these surface instabilities.

## Results

### Patterns on planar geometry

**Figure 1:**
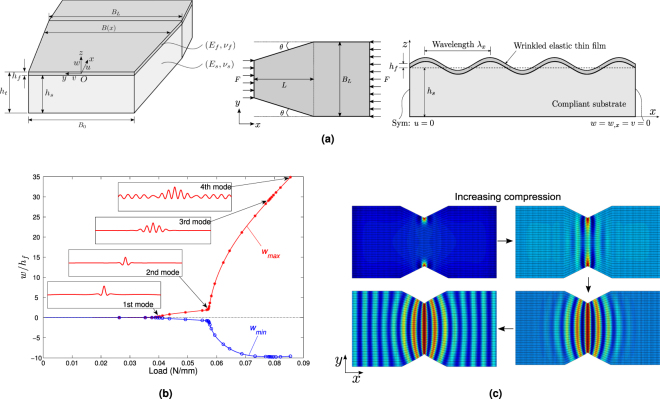
(**a**) Geometry of trapezoidal film/substrate system subjected to uniaxial compression. (**b**) Bifurcation diagram involving three bifurcations. Each point on nonlinear curves corresponds to one incremental step. Representative wrinkling modes with respect to the increasing load are marked on the post-buckling evolution path. (**c**) Top view of pattern evolution with increasing compression: corner mode, line mode, wavy mode, to wavy mode + sinusoidal mode.

We begin with planar geometry of an elastic thin film bonded to a soft substrate under uniaxial compression *F*, in the case of linearly tapered geometry with an angle tan *θ* = 0.5, as shown in Fig. 1a. Upon wrinkling, the film elastically buckles to relax the compressive stress and the substrate concurrently deforms to maintain perfect bonding at the interface. The geometric gradient leads to stress gradient that can alter uniform sinusoidal wrinkles to graded undulations where the amplitude, wavelength and direction can vary along the length together. This could be analogous to shark skin that is covered with ribbed, graded texture aligned in the streamwise direction. Let *x* and *y* be in-plane coordinates, while *z* is the direction perpendicular to the mean plane of the film/substrate. The length of the system is denoted by *L*, and the shorter and longer widths are represented by *B*_0_ and *B*_*L*_, respectively. The parameters *h*_*f*_, *h*_*s*_ and *h*_*t*_ represent, respectively, the thickness of the film, the substrate and the total thickness of the system. Young’s modulus and Poisson’s ratio of the film are respectively denoted by *E*_*f*_ and *v*_*f*_, while *E*_*s*_ and *v*_*s*_ are the corresponding material properties for the substrate. Here we consider huge modulus ratio ${E}_{f}/{E}_{s}\sim 72000$ and large thickness ratio *h*_*s*_/*h*_*f*_ = 100, which implies a stiff, thin film attached on a soft, thick substrate. Other dimensional parameters are set as *L* = 1.2 *mm*, *B*_0_ = 0.8 *mm*, *B*_*L*_ = 1.2 *mm*. The clamped boundary condition ($w={w}_{,x}=v=0$) is applied on the side *B*_*L*_, and symmetry condition (*u* = 0) is considered on the side *B*_0_ so that calculations can be performed only for the half system to reduce the computational cost. The two taper edges are set to be free. Other trapezoidal domains were studied in^39^, where similar pattern evolutions were obtained. The geometric gradient generates non-uniform distributed axial stress in the film: ${\sigma }_{f}(x)=F/[{h}_{f}B(x)]$, where the width of the system is given as *B*(*x*) = *B*_*L*_−2(*L*−*x*)tan *θ*. This gradient compressive stress leads to a localized corner mode on the shorter edges at the critical load (see Fig. 1b and c). When the load reaches the second bifurcation, the localized corner pattern tends to be a graded sinusoidal shape where the amplitude fades along the *x* direction. This wavy pattern grows and spreads along the length inside the taper region beyond the second bifurcation, while the wavelength remains almost constant. Outside the taper region where the compressive stress is uniform, straight stripes appear. The transition between the two happens at the interface where the wavelength and its amplitude alter. For the cases with a relative larger geometric gradient, wavy patterns can be constrained inside the taper region and cannot propagate outside. Hence, localized and distributed wrinkling modes are observed when the domain is trapezoidal, which is caused by non-uniform compressive stresses before the bifurcation. Particularly, the corner mode follows from stress concentration near the corner. Here the localized mode is shortly dominated by the classical 1D sinusoidal mode that further has a graded amplitude due to the stress gradient. Such mechanical response can be explained by the Ginzburg-Landau equation with variable coefficients^39^. By comparison, a uniform stress leads quickly to uniform buckling in the center, in agreement with the solution of the Ginzburg-Landau equation involving a hyperbolic tangent in the case of stable post-bifurcation behavior^40^. An iconic case of such a hyperbolic tangent envelope is a long elastic rectangular plate under uniaxial compression, as discussed theoretically in^41^ and experimentally in^42^. The graded patterns are attributed to a gradient of geometry, while similar behaviors can follow from a thickness gradient or a material gradient as in^43^, where more significant variations of wavelength have been highlighted. In the same spirit, Ginzburg-Landau equations with variable coefficients have been studied since a long time^44–46^.

**Figure 2:**
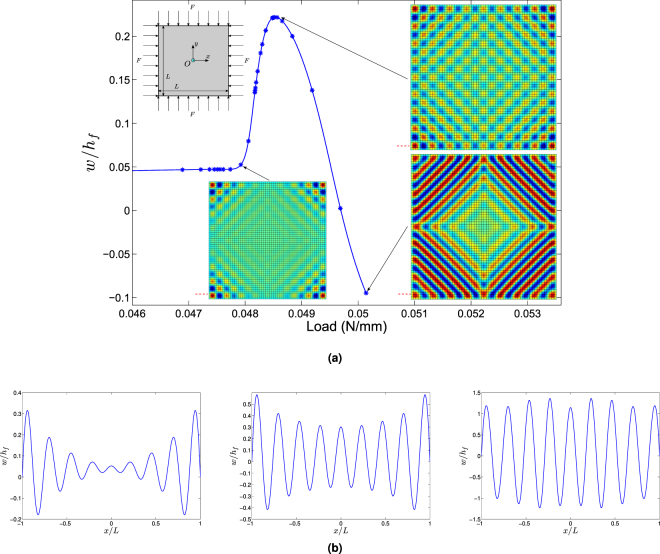
(**a**) Bifurcation diagram of the center point in a square film/substrate system under equi-biaxial compression. Two bifurcations and their post-buckling evolution are captured: a corner pattern with checkerboard shape tends to be a uniform checkerboard pattern, and finally becomes stripe patterns perpendicular to the diagonal direction. (**b**) Evolution of corresponding wrinkling modes at the cross-section near the boundary marked in Fig. 2a. Boundary mode with a hyperbolic envelope gradually evolves into a quasi-periodic sinusoidal mode with increasing load.

The second case is dedicated to a square film/substrate structure with a bigger dimension *L* = 2 *mm* under equi-biaxial compression to explore 2D short-wavelength wrinkling patterns. Simply supported boundary conditions (*w* = 0) are imposed on the four sides. Two bifurcations are found in the load-displacement curve (see Fig. 2a). The first instability mode is located in four corners with a checkerboard shape, since the equi-biaxial compression induces an isotropic compressive stress field concentrated in four corners due to boundary effects. With the increase of loading, the checkerboard patterns gradually occupy the whole domain with nearly uniform distribution. Finally the neighbouring seeds coalesce into line chains perpendicular to the diagonals that correspond to the directions of maximum principal compressive stress. The evolution of wrinkling modes of cross-section near the edge is depicted in Fig. 2b. One can observe that the boundary mode with a hyperbolic envelope gradually evolves into a quasi-periodic sinusoidal mode with increasing load. This checkerboard shape is a typical feature for planar film/substrate under equi-biaxial compression and it is similar to other wrinkling simulations in the literature^25,29^, but with a difference: the amplitude of oscillations is not uniform and is bigger close to the boundary because of extra stresses due to boundary effects (see Fig. 2).

### Patterns on curved geometry

**Figure 3:**
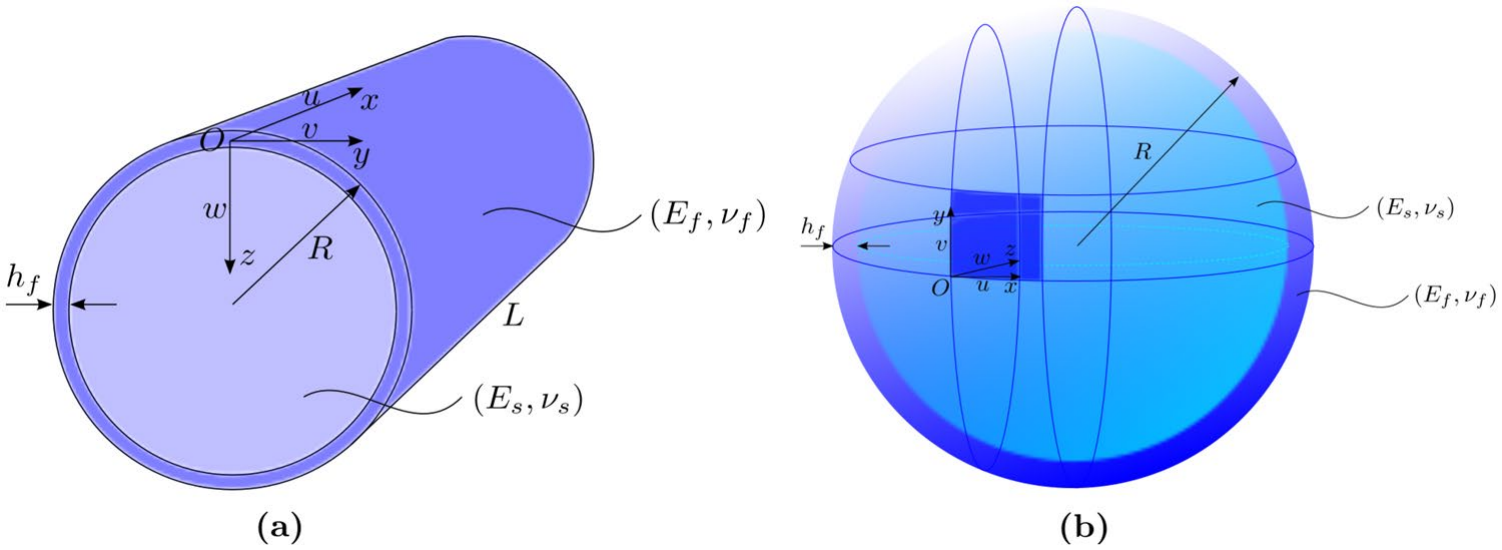
(**a**) Geometry of core-shell cylinder based on curvilinear coordinates, where *x* and *y* represent respectively axial and circumferential coordinates, while *z* the radial direction coordinate. The same frame is adapted to the components of the displacements *u*, *v* and *w*. The thickness of the shell, the radius and the length of the system are denoted by *h*_*f*_, *R* and *L*, respectively. Young’s modulus and Poisson’s ratio of the shell are respectively denoted by *E*_*f*_ and *v*_*f*_, while *E*_*s*_ and *v*_*s*_ are the corresponding material properties for the core. (**b**) Geometry of core-shell sphere. The same framework and notation as core-shell cylinder are taken into account.

**Figure 4:**
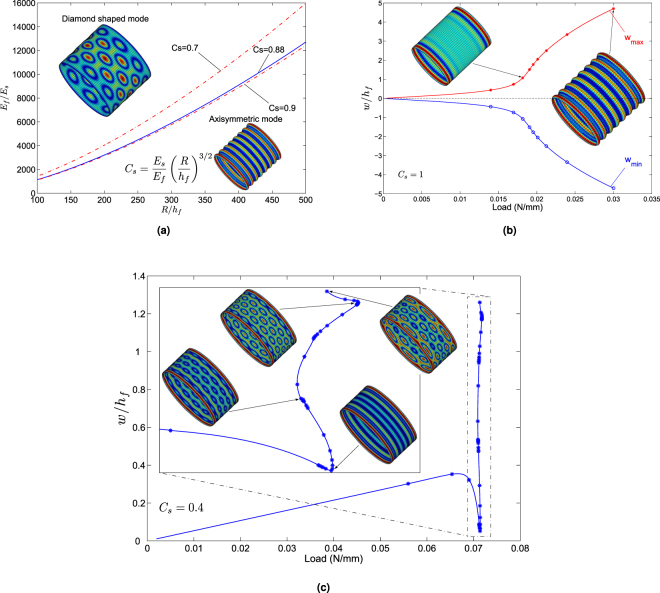
(**a**) A phase portrait on pattern selection defined by ${{C}}_{s}=({E}_{s}/{E}_{f})\,{(R/{h}_{f})}^{\mathrm{3/2}}$. The two red dash-dot curves give the upper and lower bounds obtained through numerical calculations. When the critical parameter *C*_*s*_ ≥ 0.9, the instability mode is axisymmetric; whereas at a smaller value *C*_*s*_ ≤ 0.7, the system may branch into the diamond shaped mode. The pattern selection in the narrow region 0.7 < *C*_*s*_ < 0.9 appears to be quite sensitive in the numerical results. The blue solid curve is determined by the critical parameter *C*_*s*_ = 0.88, which corresponds to the boundary defined based on Koiter’s post-buckling theory^28^. (**b**) Pitchfork bifurcation diagram of core-shell cylinder under axial compression with *C*_*s*_ = 1 and Batdorf parameter $Z={L}^{2}\sqrt{1-{\nu }_{f}^{2}}/(R{h}_{f})=455$: boundary instability mode to uniformly axisymmetric pattern. (**c**) Evolution of multiple bifurcations of core-shell cylinder under axial compression with *C*_*s*_ = 0.4: axisymmetric mode, non-axisymmetric diamond-like mode to localization mode.

Many biological structures in nature and flexible, wearable electronic devices in modern industry have curved geometric configurations to achieve certain functions as mentioned before. Recent interests are focused on harnessing curvature to tune or control surface morphogenesis with broad range of applications include adaptive aerodynamic drag control^12,20^ and microlens arrays production^11^. We will study pattern formation and evolution on core-shell cylinders and spheres, respectively, which appear to be the most representative curved geometries: cylinders have the simplest developable surface with zero Gaussian curvature, while spheres hold double curvature with positive Gaussian curvature $\kappa =1/{R}^{2}$. Coordinates, kinematics and geometries are depicted in Fig. 3. The thickness of the shell, the radius and the length of the system are denoted by *h*_*f*_, *R* and *L*, respectively. Young’s modulus and Poisson’s ratio of the shell are respectively denoted by *E*_*f*_ and *v*_*f*_, while *E*_*s*_ and *v*_*s*_ are the corresponding material properties for the core. Let us first consider an axially compressed cylindrical core-shell structure, where the radial displacement *w* is locked at both ends of the cylinder. The buckling and post-buckling pattern selection can be characterized by core stiffness measured by the dimensionless parameter ${{C}}_{s}=({E}_{s}/{E}_{f}){(R/{h}_{f})}^{\mathrm{3/2}}$ ^38^, as shown in Fig. 4a. Precisely, for a stiff core (*C*_*s*_ ≥ 0.9), the buckling pattern is axisymmetric and the post-buckling behavior is constantly supercritical and stable with a pitchfork bifurcation (see Fig. 4b); whereas for a soft core (*C*_*s*_ ≤ 0.7), multiple bifurcations are found on the complicated post-buckling response: the first two instability modes show sinusoidally deformed axisymmetric shape with boundary effects and the post-buckling evolution is supercritical. Then the bifurcated solution branch turns out to be subcritical and the associated instability modes become diamond shaped (see Fig. 4c). Gradually, the two neighbouring diamond-like patterns begin to merge into a bigger one near the boundary and this matures in the final step with a localization mode in the form of alternating deep and shallow diamond-like shapes in the circumferential direction. The classical linear shell buckling analysis^47^ predicts a number of coincident modes with short wavelength, including axisymmetric and non-axisymmetric diamond-like patterns, but the observed instability modes are usually non-axisymmetric in most of the computations and experiments for thin shells. The axisymmetric mode has only been found for thick shells in the case of buckling occurring in the plastic range^48,49^. The predominance of non-axisymmetric diamond-like modes is attributed to edge effect and hoop stresses in the boundary layer. Such boundary effect is apparent through the bulges close to the ends in Fig. 4b (see also^48^), which is generated by the mismatch between the boundary constraint and the expansion in the bulk. One finds the same scheme for a cylindrical shell with a soft substrate (see Fig. 4c), but the mode tends to be axisymmetric with a relative stiff substrate (see Fig. 4b). More surprisingly, the bifurcation curve in Fig. 4b is supercritical, which has never been found in pure shell structures.

**Figure 5:**
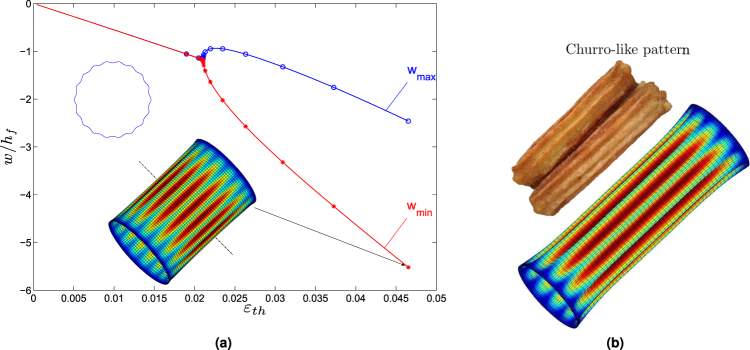
(**a**) Pitchfork bifurcation diagram of cylindrical core-shell subjected to thermal shrinkage in the core, with dimensionless parameter ${{C}}_{t}=({E}_{s}/{E}_{f}){(R/{h}_{f})}^{\mathrm{3/4}}{(L/{h}_{f})}^{\mathrm{3/2}}=266$. The post-buckling curve is supercritical and stable. (**b**) Churro-like patterns with short-wavelength instability in the circumference.

Thermal stress is the most common loading type widely existing both in nature and in industrial application. We then explore thermal wrinkling (thermal shrinkage in the core) of core-shell structures, where the clamped boundary condition ($w={w}_{,x}=u=v=0$) is employed at both ends of the cylinder. Distinguished from axial compression case, a churro-like buckling mode occurs with short-wavelength instability in the circumference and global buckling in the longitudinal direction (see Fig. 5). For a relatively stiff core with ${{C}}_{t}=({E}_{s}/{E}_{f}){(R/{h}_{f})}^{\mathrm{3/4}}{(L/{h}_{f})}^{\mathrm{3/2}}=266$, it shows a pitchfork bifurcation with a stable and supercritical post-buckling behavior. This can be explained through stress analysis: differing from uniaxial stress field ($\sigma =-\sigma {e}_{x}\otimes {e}_{x}$) in the axial compression case, thermal shrinkage will induce an isotropic stress state ($\sigma =-\sigma ({e}_{x}\otimes {e}_{x}+{e}_{y}\otimes {e}_{y})$) in the pre-buckling stage in the shell. It is well known that the critical buckling stress is generally much higher in the axial compression case than the external pressure situation, with the following orders of magnitude: $|{\sigma }_{x}^{cr}|/{E}_{f}\sim {h}_{f}/R$, $|{\sigma }_{y}^{cr}|/{E}_{f}\sim [{({h}_{f}/R)}^{\mathrm{3/2}}R/L]$. Thus, the circumferential stress σ_*y*_ would destabilize the system much earlier than the axial stress σ_*x*_ so that the corresponding instability pattern should be similar to the hydrostatic pressure loading^48,50^.

**Figure 6:**
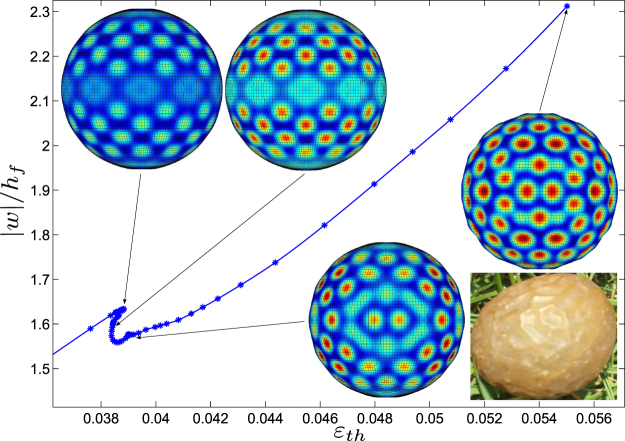
Snap-back bifurcation portrait of core-shell sphere subjected to thermal shrinkage in the core, with dimensionless parameter ${{C}}_{s}=({E}_{s}/{E}_{f}){(R/{h}_{f})}^{\mathrm{3/2}}=5$. Dimple patterns gradually evolve into buckyball-like modes consisting of regular pentagons and hexagons, which is analogous to dehydration of core-shell fruits in a dry environment.

Thermal wrinkling of a spherical shell supported by a core (*C*_*s*_ = 5) shows totally different pattern formation and evolution behavior, which involves dynamic movement, rotation, and coalescence of polygons formed in the post-buckling stage (see Fig. 6). When the thermal shrinkage reaches a critical value, the sphere suddenly bifurcates into a periodic dimple structure, and then evolves into buckyball-like pattern consisting of regular pentagons and hexagons, with a snap-back post-buckling response. This thermal shrinkage can be equivalent to dehydration of core-shell fruits in a dry environment and one can observe polygonal patterns on the exocarp. Quantitative understanding the post-buckling evolution and morphological transition of core-shell structures is not only beneficial for applications in biomedical engineering but also gives a potential fabrication route to multi-functional surfaces.

All these deformed shapes depend mainly on loading and geometry, rather than the presence of the substrate. Indeed, in the examined three curved shells, patterns appear to be the same with or without substrate, except for the very special case of the axisymmetric mode for an axially compressed core-shell cylinder. The main contribution of a relative stiff substrate is the stabilization of the post-bifurcation path, which has been observed for cylinders under various loads^28,38,51^. In the case of spherical core-shell, bifurcations remain subcritical, probably because the spherical symmetry induces a bi-dimensional kernel^52^. Nevertheless, the hysteresis loop appears to be rather narrow. Hexagonal patterns are quite common for convective instabilities^45,53^, for the buckling of pressurized spherical shells^49^ or spherical core-shells^12^ as considered here. In the latter paper, the physical/numerical model is similar to the one applied in this work, but with a high simplification for the substrate. Here we consider a full 3D description of the substrate. Stoop *et al*.^12^ present almost perfectly periodic patterns that degenerate into more or less disordered structures, while the wrinkling modes in Fig. 6 are neither strictly periodic, nor highly disordered. Likely the three types of patterns (periodic, ordered but not periodic, disordered) can exist for systems with a spherical symmetry. These three morphologies occur in the simulations of a toroidal geometry as well^12^.

## Discussion

### Loading effect

Wrinkling patterns strongly depend on loading type that can generate different orientations of compressive stresses. As for planar film/substrate systems, uniaxial compression leads to 1D sinusoidal surface wrinkles perpendicular to the loading direction (see Fig. 1), while equi-biaxial compression can induce symmetric 2D checkerboard patterns (see Fig. 2a). Nevertheless, with the increasing of compression, localized maximum principal compressive stress can alter the patterns from checkerboard to sinusoidal reversely following diagonal directions. At the pre-buckling stage, boundary effects are observed in the biaxial compression case due to stress concentration in four corners.

As for core-shell cylinders, pattern formation and evolution under axial compression and thermal shrinkage are totally distinguished from each other (see Figs 4 and 5), which is mainly due to different stress distributions in the pre-buckling stage as explained before. Under axial compression with uniaxial stress field, the buckling mode can be axisymmetric or non-axisymmetric diamond shaped beyond secondary bifurcations; whereas for thermal shrinking with biaxial stress state, it always shows churro-like patterns with short-wavelength instability in the circumference but global buckling in the longitudinal direction.

### Localization and boundary effects

Boundary value problems as considered in this paper generally have boundary effects. For the case of equi-biaxial compression of planar film/substrate systems as shown in Fig. 2a, the first bifurcation pattern turns out to be a boundary instability mode, since the compression leading to stress concentration around four corners can first destabilize the film therein. This boundary effect can also be observed in curved structures, namely core-shell cylinders under axial compression as shown in Fig. 4b. Significant boundary layers appear near both ends at the pitchfork bifurcation, which can be characterized by the famous Batdorf parameter $Z={L}^{2}\sqrt{1-{\nu }_{f}^{2}}/(R{h}_{f})=455$ ^50^. In practice, this number is often large (*e.g. Z* ∼ 500). When it becomes quite small (*e.g. Z* is in the range 20∼50), the influence of the boundary conditions extends to the whole structure, while this effect is constrained to the boundary layers for large values of the Batdorf parameter (*Z* ≥ 100)^38,51^. Nevertheless, the influence of these boundary layers diminishes in the post-buckling stage as shown in Fig. 4b.

The use of finite element method allows accounting for boundary conditions, which is not possible by the widespread Fourier approach^34–36^. Indeed, we have obtained apparent boundary effects, except for the sphere that is edgeless. In the first two cases, *i.e*. trapezoidal and square film/substrate systems, boundary effects are limited to the neighborhood of the first bifurcation, and the localized patterns disappear rapidly and are gradually replaced by a nearly periodic pattern. The axially compressed cylinder is quite specific because of a large number of bifurcation modes. In this case, boundary effects favor the diamond-like mode, but only for a soft core (*C*_*s*_ ≤ 0.7). For the thermally loaded cylinder, the axial modal profile and the critical load depend on boundary conditions, since there is only one half wave in the axial direction^51^.

The localization by edge effect should not be confused with the localization due to subcritical bifurcation in large domains. In this case, the Ginzburg-Landau equation has a solution involving a hyperbolic secant (instead of a hyperbolic tangent for a supercritical bifurcation), which spontaneously leads to localized patterns^54^. There exist important works^55,56^ on this type of localization inside the domain, resulting in complicated response curves. A number of localized and stable patterns can be created, typically for variants of the Swift-Hohenberg equations^57,58^. In our computations, we have not observed such localizations in the domain, likely because the bifurcations are often supercritical or weakly subcritical in the considered film/substrate systems. Note that there might exist several post-critical solutions even in supercritical cases^41,59^, but they are not easily captured by a path-following algorithm.

### Gradient effect

Geometric gradient can change wrinkling profile to create ribbed and graded structural patterns with variation of wave direction, amplitude and wavelength together (see Fig. 1). These ribbed and graded patterns are caused by stress gradient stemming from irregular geometric topology^39^. In fact, graded wrinkles are not straight stripes but hold a wavy curvature shape that is perpendicular to the trapezoidal edges due to the release of stresses on the boundary. The pattern evolution could be used to guide the design of geometrically gradient film/substrate systems to achieve the desired wavy instability patterns.

### Curvature effect

Topological constraints of curved surfaces play an important role on instability pattern formation and mode selection. When planar film/substrates are bent with a curved surface, *i.e*. core-shell cylinders or spheres, the buckling and post-buckling behavior are significantly altered. As for the core-shell cylindrical structure under uniaxial compression, buckling and post-buckling behavior mainly depends on the core stiffness measured by the dimensionless parameter ${{C}}_{s}=({E}_{s}/{E}_{f}){(R/{h}_{f})}^{\mathrm{3/2}}$ that involves curvature effect from the second term (see Fig. 4a). Precisely, for a stiff core (*C*_*s*_ ≥ 0.9), the buckling pattern is axisymmetric and post-bifurcation solutions are stable (see Fig. 4b); whereas for a soft core (*C*_*s*_ ≤ 0.7), the bifurcated solution branch is often subcritical and the associated instability modes are diamond-like beyond secondary bifurcations (see Fig. 4c). This suggests that the uniaxial compression can lead to 2D instability modes due to the curvature effect in core-shell cylindrical systems. The phase diagram in Fig. 4a could be helpful for morphological design of core-shell surface patterning.

Thermal wrinkling patterns are totally different between spheres and cylinders even though both have curved surface, the former holding a positive Gaussian curvature while the latter having a zero one. Spheres exhibit dimple to buckyball-like mode transition, with double periodicity both in longitude and latitude. In contrast, cylinders undergo circumferential local buckling and global buckling along the axial direction. These differences are attributed to distinguished geometric curvatures.

## Methods

### Model

Quantitative predictions of diverse wrinkling patterns were carried out based on a nonlinear finite element model, which was first established for planar film/substrate systems^29^ and subsequently extended to hyperelastic constitutive laws^60^ as well as non-planar geometry such as core-shell cylindrical systems^38^. This finite element framework appears to be versatile for both planar and curved configurations as well as various loads. In this model, the surface layer is represented by a thin shell model to allow large rotations while the core is modeled by small strain elasticity. Indeed, the considered morphological instabilities are governed by nonlinear geometric effects in the stiff material on surface, while these effects are much smaller in the soft material for the substrate. A thorough investigation on comparison between finite strain hyperelastic material model and small strain Hooke’s elasticity, with respect to a wide stiffness range of Young’s modulus was performed in^60^. The limit of large/small strains can also be approximated through the critical buckling load for 1D sinusoidal wrinkles^61^, *i.e*. ${\varepsilon }_{cr}=\mathrm{1/4}{(3{\overline{E}}_{s}/{\overline{E}}_{f})}^{\mathrm{2/3}}$, where ${\overline{E}}_{f}={E}_{f}/(1-{{\nu }_{f}}^{2})$ and ${\overline{E}}_{s}={E}_{s}/(1-{\nu }_{s}^{2})$. If considering *ε* ∼ 5% as the upper bound of small strain, then one can obtain modulus bound *E*_*f*_/*E*_*s*_ ∼ 30. Precisely, for a stiffer film with ${E}_{f}/{E}_{s} > 30$, the critical buckling strain is lower than 5% so that Hooke’s law is relevant. Therefore, in most cases of film/substrate systems, *i.e*. ${E}_{f}/{E}_{s}\gg {\mathscr{O}}\mathrm{(30)}$, linear constitutive laws appear to be sufficient and are qualitatively or even quantitatively equivalent to finite strain hyperelastic models^60^.

Buckling and wrinkling instabilities are mainly induced by compressive stresses, leading to the decrease of the tangent stiffness of systems. This loss of stiffness being proportional to the current stress state, its effect is much more significant in the film. That is the reason why it is proposed in^61^ to model the substrate with linear elasticity while the constitutive law of the film is expressed in terms of the nonlinear Green-Lagrange strain tensor according to Saint-Venant Kirchhoff model, and this framework is maintained here, which means the strain energy in the film is a quadratic function of the Green-Lagrange strain tensor, *i.e*. $\mathrm{1/2}\gamma :{{\bf{L}}}_{f}:\gamma$, where **L**_*f*_ is the elastic tensor of the film. Due to the thinness of the film, a shell model is highly recommended and a number of finite elements are well established to achieve this discretization, for instance in commercial software Abaqus^62^. Our computational scheme is based on nonlinear shell elements introduced in^63^ and its robustness has been validated for nonlinear elastic thin-walled structures such as cantilever beam, square plate, cylindrical roof, circular deep arch^64^, and planar^29^ or curved^38^ film/substrate systems. Each element is a curved quadrilateral with 8 nodes and 48 degrees of freedom (DOFs).

Linear isotropic elasticity theory can accurately describe the substrate. Hence, the potential energy of the substrate can be expressed as

1 $${{\mathscr{P}}}_{s}({{\bf{u}}}_{s})={\int }_{{{\rm{\Omega }}}_{s}}\frac{1}{2}({}^{t}{\varepsilon }:{{\bf{L}}}_{s}:{\varepsilon }-{}^{t}{\varepsilon }:{{\bf{L}}}_{s}:{{\varepsilon }}_{th})d{\rm{\Omega }},$$

where **L**_*s*_ is the elastic tensor of the substrate. The total strain and thermal strain are respectively denoted as *ε* and *ε*_*th*_. Here 8-node linear brick elements with reduced integration are applied to discretize the substrate, with totally 24 DOF on each brick element. In the cases where the substrate is subjected to the thermal shrinkage, the thermal strain can be expressed as

2 $${\varepsilon }_{th}=\alpha {\rm{\Delta }}T{\bf{I}}\,{\rm{with}}\,{\rm{\Delta }}T < \mathrm{0,}$$

where *α*, Δ*T* and **I** denote the thermal expansion coefficient, temperature change and second-order identity tensor, respectively. This thermal shrinking loading ***ε***_*th*_ can be characterized by a residual strain *ε*_*th*_= *ε*_*res*_ = −*λ* **I**, where *λ* is a scalar load parameter and only normal strains are considered for isotropic loading.

As the surface film is bonded to the substrate, the displacement should be continuous at the interface. Shell elements and 3D brick elements, however, cannot be simply joined directly since they belong to dissimilar elements that hold different displacement degrees of freedom. Hence, additional incorporating constraint equations have to be employed and here Lagrange multipliers are applied to couple the corresponding nodal displacements in compatible meshes between the shell and the substrate. Consequently, the stationary function of the core-shell system is given in a Lagrangian form:

3 $$ {\mathcal L} ({{\bf{u}}}_{f},{{\bf{u}}}_{s},\ell )={{\mathscr{P}}}_{f}+{{\mathscr{P}}}_{s}+\sum _{node\,i}{\ell }_{i}[{{\bf{u}}}_{f}(i)-{{\bf{u}}}_{s}(i)],$$

in which ${{\mathscr{P}}}_{f}$ represents the potential energy of the film. The displacements of the shell and the core are respectively denoted as **u**_*f*_ and **u**_*s*_, while the Lagrange multipliers are expressed by $\ell$.

### Resolution

A path-following continuation technique named *Asymptotic Numerical Method* (ANM)^65^ is applied to solve the resulting nonlinear PDEs (Eq. 3). The ANM is a numerical perturbation technique based on a succession of high-order truncated power series with respect to a well chosen path parameter, which appears as an efficient continuation predictor without any corrector iteration. It gives interactive access to semi-analytical equilibrium branches, which offers considerable advantage of reliability compared with classical iterative algorithms. Besides, one can get approximations of the solution path that are very accurate inside the radius of convergence. By taking advantage of the local polynomial approximations of the branch within each step, the algorithm is remarkably robust and fully automatic. Furthermore, unlike incremental-iterative methods, the arc-length step size in the ANM is fully adaptive since it is determined *a posteriori* by the algorithm. Here the main interest and advantage of the ANM lie in the ability to trace the post-buckling evolution on the equilibrium path and to predict secondary bifurcations without any special tool. Precisely, accumulation of small steps in the ANM is often associated with the occurrence of a bifurcation^29,66^.
